# HB-HTA as an implementation problem in Polish health policy

**DOI:** 10.1371/journal.pone.0257451

**Published:** 2021-09-24

**Authors:** Małgorzata Gałązka-Sobotka, Aldona Frączkiewicz-Wronka, Iwona Kowalska-Bobko, Hanna Kelm, Karolina Szymaniec-Mlicka

**Affiliations:** 1 Institute of Healthcare Management, Lazarski University, Warsaw, Poland; 2 Department of Public Management, University of Economics in Katowice, Katowice, Poland; 3 Faculty of Health Science, Institute of Public Health, Medical College, Jagiellonian Univeristy, Krakow, Poland; University of Adelaide, AUSTRALIA

## Abstract

Hospital Based Health Technology Assessment (HB-HTA) is a new policy implemented in Poland to allow for a more practical and contextualized assessment related to the use of specific medical procedures, devices, or equipment. It requires changes in governance relating to the healthcare sector. One of the forms of governance improvement is to involve society in the process of creating public services. This can be implemented, e.g., by applying the pragmatic model of public responsiveness. The aim of this research was to identify and analyze forces which will shape a dynamic process in determining the implementation of HB-HTA. The results obtained in the Gioia analysis led to the identification of the main forces driving and restraining the implementation of HB-HTA. The grouping and interpretation allowed for the twelve most important dimensions to be distinguished, which were recognized as conceptual categories necessary to build theories that describe the studied phenomenon. This study contributes to the development of the idea of responsiveness in public management theory and in health care services, and ultimately helps to better enable the adjustment of health services to the dynamically changing needs of Polish society.

## Background—Responsiveness in the perspective of HB-HTA implementation process in Poland

Change is a “broad phenomenon that involves the growth and development of one or more of a number of elements of a public service” [1]. According to Brown and Osborne a change may include designing a new service, restructuring the institution providing public services, redesigning management and administration of this institution, or gaining skills required for providing and managing public services.

There are many reasons people resist change. Change means loss of control, uncertainty, and quite often, more work to be done, which may lead to responsibility overload. Change may also be considered distracting or confusing, interfering with peoples’ activities and causing disruption. People may have concerns about their abilities and whether they have the adequate skills to handle new circumstances [2].

Considering the above-mentioned reasons, when planning a change in the provision of public services, one should carefully examine whether a given reform will be met with favor or rejection by the people and institutions involved. A change in public policy should be well-thought-out and prepared, and this requires the use of specific instruments.

Part of preparing for a change in public policies is the search for support forces in the process of new implementation in public management. In the case under study, there is a planned change in health policy, namely, the implementation of Hospital-based Health Technology Assessment (HB-HTA). HB-HTA is based on performing health technology activities tailored to the hospital context for managerial decisions.

Health Technology Assessment (HTA) facilitates decision-making by legislative bodies on the basis of reliable scientific research and economic analysis of the circumstances of a particular healthcare system [3]. According to the EUNetHTA, a health technology assessment organization network in the European Union, HTA is a:

*“multidisciplinary process that summarizes information on medical*, *social*, *economic and ethical issues related to the use of a given health technology in a systematic*, *transparent*, *impartial and robust manner*. *Its purpose is to provide the information needed to create safe and effective patient-centered health policies and a desire to achieve the best value”*

[4].

Health technology assessment does not have to refer only to drug technologies and to the functions of large national health technology assessment agencies. Usually, medical facilities need more practical assessment of different specific procedures or equipment in hospital units. Hence the concept of hospital health technology assessment (Hospital-Based Health Technology Assessment, HB-HTA), which applies tools used in health technology assessment processes (including budget impact analysis and pharmacoeconomic analyses) in the context of the functioning of individual hospitals. The main assumption for the implementation of health technology assessment at the hospital level is that there are hospitals that are introducing modern technologies, and to implement HTA, they need proper evaluation of the given procedures and evidence for their application [5].

In order to effectively support the implementation of HB-HTA, the Polish government finances the GOSPOSTRATEG program, as part of the National Center for Research and Development activities, in accordance with the recommendation of the Council of the European Union and the European Innovation Partnership on Active and Healthy Aging (EIP-AHA), thus improving the self-sufficiency and performance of healthcare systems through the use of objective health technology assessment methods that aim to identify effective health technologies for clinical practice application under national healthcare systems.

Highly developed countries are constantly looking for more effective and efficient forms of public management. A key stage of change in this area was the adaptation of the New Public Management and Governance concepts. This involves the absorption of market mechanisms as well as management methods and techniques widely used in the private sector into the public sector, as well as the administration’s focus on performance, economic efficiency, quality, and result orientation. Although the New Public Management and Governance implementation process has been going on for several decades, it still requires continuous improvement. First, it should be understood that the public sector cannot directly copy market mechanisms because there are some fundamental differences between free market customers and public service recipients. The latter have many more inalienable rights that customers do not have [6].

One of the forms of improving public management is to involve the public in the process of creating public services. It can be implemented, e.g., by using the idea of responsiveness. Generally, responsiveness "denotes the *speed* and *accuracy* with which a service provider responds to a request for action or information" [7]. According to Ostrom, public responsiveness reflects "the capacity to satisfy the preferences of citizens" [8]. The subject of responsiveness in healthcare has been discussed in recent years by, among others, Forouzan et al. (2016) [9], Chao et al. (2017) [10], Daneshkohan et al. (2018) [11], and Hamid and Begum (2019) [12].

Social or bureaucratic responses to citizens’ demands are crucial in public administration theories. Responsiveness has become a key concept regarding the proper role of bureaucracy and professional administrators in a democratic political system. At the local level, reactivity to public needs is particularly important, given that local experts have constant and direct contact with residents. In terms of public administration, responsiveness means quickly and accurately diagnosing the needs of citizens and providing public services that respond to these needs. As Fried states “responsiveness is an important value for government organizations” [13]. Public responsiveness is also an important criterion, together with effectiveness and liberalism, for evaluating government performance [14–17].

In the literature, some attempts to structure the concept of responsiveness may be found. Schumaker [18] defined five basic forms of responsiveness: access responsiveness, agenda responsiveness, policy responsiveness, output responsiveness, and impact responsiveness. Bryer [14] defines six variants of administrative responsiveness in contemporary democracy: dictated responsiveness to elected officials, constrained responsiveness to rules/norms/procedures, purposive responsiveness to administrator-defined goals, entrepreneurial responsiveness to individual customers, collaborative responsiveness to stakeholder consensus, and negotiated responsiveness to conflicting demands.

Based on previous research, Liao [6] introduced three models of responsiveness, namely: (1) the citizen-driven model, (2) the expertise-driven model, (3) the pragmatic model of public responsiveness.

They are distinguished based on the role that administrators play in pursuing public responsiveness, the behavioral norms that administrators need to obey in pursuing public responsiveness, and the goal of public responsiveness.

The citizen-driven model assumes that public administration, fulfilling its democratic responsibilities, should meet the needs of society [19]. In this model, public administration plays a subordinate role to both society and public authorities.

In practice, the citizen-driven model of public responsiveness has its weaknesses. Public administration cannot function only as an institution that fulfills the wishes of society [20], especially since different social groups may report different, often conflicting needs.

HB-HTA procedure also responds to many social healthcare needs. In general, society expects medical services that are available (preferably, universal), high quality, and in the case of the Polish healthcare system, without additional costs, except for compulsory health insurance contributions. In addition to these needs, society also expects that the treatment methods used will be innovative and effective. HB-HTA allows hospitals to implement innovative medical technologies to suit the needs of the local population, increasing the quality and effectiveness of services provided.

The necessity to evaluate the effectiveness of health technologies has increased over the past decade, particularly in patient care facilities, mainly due to their limited resources and the continuous stream of innovative, promising technologies emerging daily. Nowadays, hospitals, especially large units, use various health technologies, especially drugs, but also medical devices and equipment. Innovative medical technologies also include therapeutic and diagnostic procedures as well as changes in the system of organization of health care delivery. Decision making regarding the implementation of health technologies has traditionally been based on such issues as: clear medical trends, institutional prestige, professional pressures, expert advice and efforts or budget viability. Increasing costs of health care and rapid technological progress create the need to apply a holistic, multidisciplinary and, above all, scientific approach to the implementation of innovative health technologies. This improves the decisions’ rationality and efficiency while minimizing opportunity costs. By bridging the “know-do gap” between research and practice, HTA creates a bridge between the world of science and decision making practice in hospitals [21].

The second responsiveness model, the expertise-driven model, assumes that there should be another element between social expectations and reactive public administration activities, namely professional expertise. It is with the help of expert knowledge that real social needs are defined [22,23], because society is not able to define its needs in a holistic way with a broad perspective. Experts should also teach society to identify and name their real needs and to align them with the needs of the general public, not just their own social group [6].

In the expertise-driven model, the last word regarding public policy is the responsibility of experts, and they decide what form responsiveness is going to take, even if, in the end, the action will be different from the original expectations of society. This model of responsiveness, however, can be accused of too little political sensitivity to social needs.

Public responsiveness could be achieved no matter if it is the result of citizen preferences coming into line with existing policy, or policy reacting to citizen preferences [24]. In the case of introducing the HB-HTA procedure, we are dealing with policy reacting to citizen preferences.

The third model, the pragmatic model of public responsiveness, tries to cope with the weaknesses of the previous two by combining social expectations and expert knowledge in the process of implementing public policies. This model of responsiveness modifies the needs articulated by society in a practical way, introducing processes and procedures to achieve the desired goals.

The pragmatic model of public responsiveness best corresponds to the method whereby HB-HTA procedure has been introduced in the Polish healthcare system because this model assumes that public administration authorities take responsibility for the local community. In Poland, the owners of public hospitals are primarily local government units, which, as public administration units, are responsible for management and the organizational and infrastructural standards created in their subordinated units [25].

The introduction of HB-HTA enables hospital managers to make rational decisions on the implementation and use of innovative medical technologies, which also means that they will be able to better tailor their services to local needs, and thus take responsibility for the well-being of their patients. According to Yang and Pandey, “empowered employees are more likely to take initiative and respond to the changes of environment and citizen preferences” [20]. Therefore, hospital health technology assessment should be carried out based on scientific evidence but implemented into the reality of hospital environments.

Hospital-based HTA consists in contextualizing HTA according to the needs of that particular hospital. This attitude considers the local organizational context in which the health technology is being adopted, such as available comparators (treatment currently used locally) or available resources.

Society expects high-quality, innovative social services, and in the analyzed case, health services. HB-HTA is a response to these expectations by giving hospitals the opportunity to build their own technology profile.

## Methods

In order to correctly identify determinants of the HB HTA implementation process in the Polish healthcare system, we familiarized ourselves with the results of a study in which purposefully selected publications from the Medline (via PubMed), EMBASE, AdHopHTA, European Observatory on Health Systems, Healthcare Systems in Transition, and the WHO database were analyzed [26].

The selective literature review aimed to analyze organization and HB-HTA models in several countries, identify interactions between entities involved in the HB-HTA process, characterize stakeholders critical to the success of the HB-HTA solution (specifically identifying their roles and significance), and describe the relationships between entities (on supranational, national, regional levels) cooperating in processes related to the implementation of HB-HTA. The literature review, as we mentioned above, was supplemented with the analysis of the results of four in-depth interviews conducted with the participants of the AdHopHTA [26]. The purpose of the interviews with the AdHopHTA project partners from Austria, Denmark, France and Spain was to address the issue of the sustainability of HB-HTA solutions, the scope of modifications, the standards adopted as part of intra- or inter-institutional HB-HTA related measures, stakeholders driving and restraining the implementation and functioning of HB-HTA, the evaluation of the efficacy of the national healthcare system following the implementation of HB-HTA, and potential directions for the development of HB-HTA. The information obtained from the sources indicated above supported us in further considerations.

The aim of our study was to identify and analyze forces which will shape a dynamic process in determining the implementation of HB-HTA. Our study reflects the interpretive research tradition [27] in qualitative research, where the essence of phenomena is constructed by individuals taking part in the processes studied.

**Fig 1:**
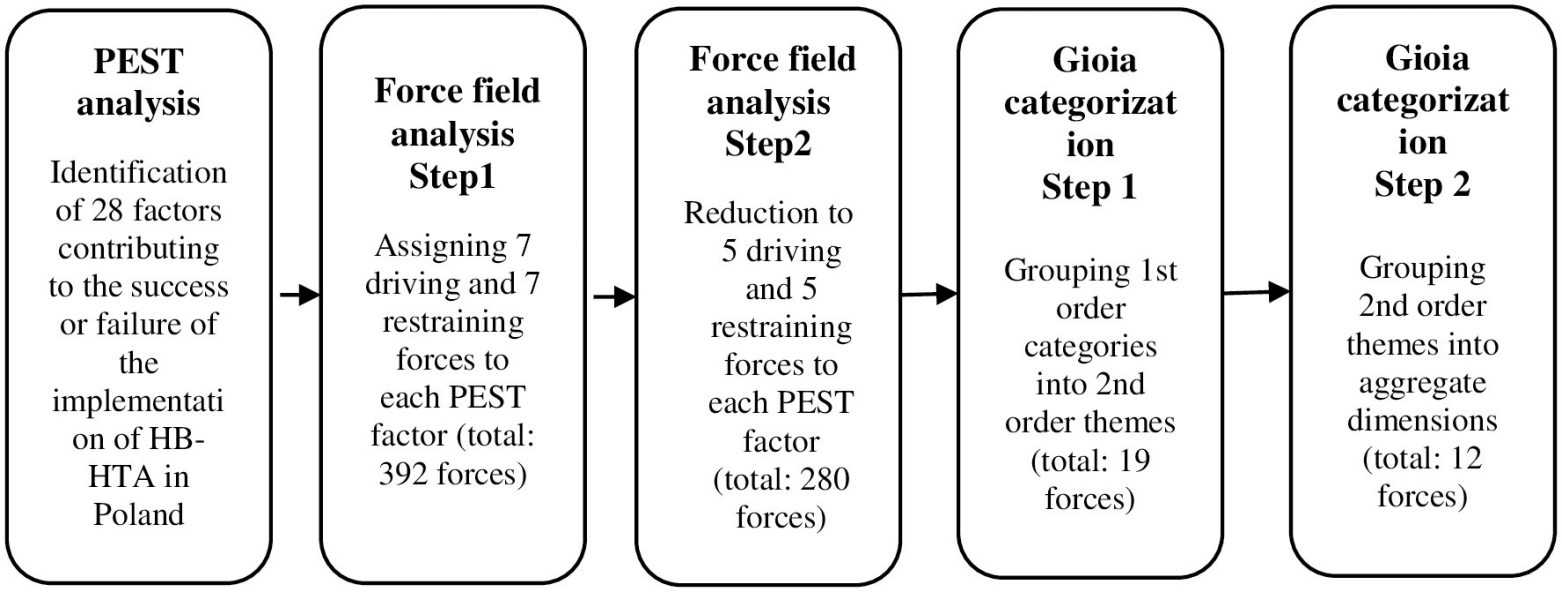
Research design.

The chart below (see Fig 1) shows the main steps that led to the identification of forces driving and restraining the implementation of HB-HTA in Poland and the methods that were used during those steps to obtain data.

The pursuit of answers to the research problem concerning the identification and nature of the main forces affecting the process of implementing HB-HTA in the Polish healthcare system required an exploratory research strategy, typical of grounded theory [28–31]. Studies conducted under grounded theory, which uses inductive reasoning and the assumptions of symbolic interactionism [32–35], require the researcher to follow the principles defined in the literature [36–38].

According to Gephart [27] qualitative research, manuscripts that emerge from broad, ongoing research programs seem more likely to produce substantial new insights because they address multiple issues and have large corpora of data to analyze.

To obtain data, the study applied the triangulation [39] of methods (combining different research methods), data (the use of the results of surveys conducted in different research groups, at different times and in different places) and researchers (information gathered by many researchers). In social systems both the problem and the corresponding solution emerge gradually [40], so the research assumptions of the study were discussed during field research and extended or reduced at every stage of data collection. Data collection included the synthesis of regular literature review, the analysis of transcribed in-depth interviews and focus group discussions, and the analysis of organizational documents. As Glaser and Strauss [41] stated, it was a work- and time-intensive process, but it was necessary to create a thick description [42], which allows for the building of the possibly most complete picture of the studied phenomenon.

The process of constantly comparing collected fragments of empirical material is very useful for the exploratory purpose of identification and researching the nature of forces that will affect the process of implementing HB-HTA in the Polish healthcare system. It is a method of analyzing quantitative data which offers a “systematic approach to new concept development and grounded theory articulation” and brings “qualitative rigor” and transparency to conducting and presenting inductive research. In the presented study, the Gioia approach is used to thematize and aggregate the identified factors conducive to the implementation of HB-HTA in the Polish healthcare system.

The selection of research samples was carried out in accordance with the proposal of Rorty [43], who argued that first the researcher should ask himself what the objective of the study is and then he should adapt the research approach to this objective and choose the sources that will help him achieve it. Data collection in research conducted according to the principles and rules of grounded theory involves creating such a sample that will generate as diverse research material as possible [31]. The application of the principles of grounded theory at the beginning of the study means that we know neither the ultimate size nor the composition of the sample. More objects were added in the process of inquiry to obtain a broad spectrum of data on the studied phenomenon. The data collection process lasted until theoretical saturation was achieved, which means that further data collection would neither enrich our knowledge about the studied phenomenon nor further contribute to the theory we are developing [44].

### Contextual analysis using PEST analysis and Force Field analysis

#### PEST analysis

The first stage of our research was identifying the determinants for implementing HB-HTA in Poland. Macroenvironmental determinants of the implementation of HB-HTA were investigated using strategic PEST analysis. This involves defining the basic areas of the environment, i.e., the areas that can have a key impact on an organization and its future strategy. The aim of the analysis was to identify political, economic, social, and technological factors that may contribute to the success or failure of the implementation of HB-HTA. PEST analysis is a powerful and widely used tool (also in public management) for identifying the most important factors in macroenvironment which affect the possibility of implementing a new concept, product, or service [45]. PEST is an acronym for four sources of change: political, economic, social, and technological. “Political factors (P): these cover various forms of government interventions and political lobbying activities in an economy. Economic factors (E): these mainly cover the macroeconomic conditions of the external environment but can include seasonal/ weather considerations. Social factors (S): these cover social, cultural, and demographic factors of the external environment. Technological factors (T): these include technology related activities, technological infrastructures, technology incentives, and technological changes that affect the external environment” [46].

PEST factors were identified in the process of the analysis and synthesis of focus group interviews with 50 selected experts (purposive sampling) consisting of (a) representatives of healthcare system entities in Poland, (b) health care service providers, (c) payers’ institutions, (d) professional associations, (e) patient associations, (f) regional and local authorities, (g) hospital management, and (h) hospital staff. The experts were selected based on an assessment of their past achievements in the management of health care entities. An invitation to participate in the study was sent to each of the selected experts, which explained in detail the objectives of the study and the scope of work envisaged for the expert. After granting their permission to participate in the study, the expert was asked to agree in writing to participate in the study and to maintain confidentiality and not to disseminate the results of the study beyond the official circuit including reports, conference papers until the final conclusion of the work in the GOSPOSTRATEG project and the posting of all its results on the platform www.hbhta.pl.The focus meetings (which took place at Lazarski University in Warsaw) with the experts were conducted by four moderators. There are many potential difficulties in conducting focus groups, such as a poorly prepared moderator, difficulties encountered in group dynamics, an underdeveloped scenario, the passivity of the presenter, and the inability to focus the discussion on a particular issue. These can all be causes for the loss of control over the course of the meeting and have a major impact on the quality of information obtained [47]. Loss of control over the discussion prevents the key problems of the study as written in the scenario from being realized. In any group discussion, the varying characters of the participants become apparent at different times. Some are more compliant and calmer, while others are dominant and expansive both in terms of influencing the views of others and in terms of non-verbal communication. In the research described here, the moderators’ role was to control group dynamics, while not being indifferent to receiving responses from diverse interlocutors. The moderators in the initial phase of the meeting tried to recognize the profiles of the respondents and, in accordance with their personalities, choose the strategy of using verbal and non-verbal messages. The large flexibility in choosing the appropriate tactics during the focus group research was due to the accumulation of knowledge and experience of the moderator [48].

The moderators of the work on the identification of political, economic, social, technological determinants were people involved for many years in the process of reforming the health care sector in Poland and were well known in the professional and scientific community. The moderators were obliged to give written consent to participate in the research and for confidentiality in terms of dissemination of the research results. For the sake of scientific objectivity, the moderators did not provide experts with their opinions on the issues examined. Meetings were conducted according to a set scenario, and experts received a list of questions. The first round of meetings included four parallel focus interviews in groups working to identify the political, economic, social, and technological determinants of the implementation of HB-HTA. In the second round of the study, the participants were acquainted with the summary results of the first round of focus interviews. The participants also assessed and verified the factors characterizing the political, economic, social, and technological environment proposed by the panel moderators. Sample size, moderators and participants presented in Table 1.

**Table 1:** Sample size and moderators.

Environment (moderator)	Number of participants (experts)	Participants
Political (Prof. Iwona Kowalska-Bobko)	11	representatives of the National Health Fund (NHF) representatives of the Agency of Health Technology Assessment and Tariff System (AOTMiT) management staff from hospitals, sectoral employees
Economic (PhD Małgorzata Gałązka-Sobotka)	12	representatives of the National Health Fund (NHF) representatives of AOTMiT representatives of the Ministry of Health management staff from hospitals, sectoral employees
Social (Krzysztof Łach)	16	representatives of the National Health Fund (NHF) representatives of AOTMiT management staff from hospitals, sectoral employees representatives of patient organizations
Technological (PhD Iwona Skrzekowska-Baran)	11	representatives of the National Health Fund (NHF) representatives of AOTMiT management staff from hospitals, sectoral employees representatives of patient organizations HTA specialists

In the work on the synthesis of the obtained results, the moderators were additionally supported by 8 researchers of the health sector problems selected through the evaluation of the documentation confirming their scientific achievements submitted to the competition announced by the coordinator of the GOSPOSTRATEG project. 12 researchers (including 4 moderators) analyzed the results of focus meetings. As a result, the PEST analysis identified 28 factors characterizing the relevant environments (see Table 2).

**Table 2:** Results of the PEST analysis.

Political factors: (a) building a political agenda, (b) regulatory capabilities, (c) implementation possibilities, (d) involvement of central institutions in promoting the introduction of HB-HTA into the healthcare system, (e) involvement of local and regional institutions in promoting the introduction of HB-HTA into the healthcare system, (f) hospital independence, conditions, and adaptability, (g) monitoring and evaluation.	Economic factors: (a) expenditure on health care, (b) the share of public expenditure on health care of total health expenditure, (c) the share of expenditure on salaries of the total hospital costs, (d) costs of medical technologies, (e) availability of European funds for investments in innovative medical technologies, (f) flat-rate financing of hospitals, (g) competence of hospital management in HB-HTA analysis skills.
Social factors: (a) patient participation in the process of assessing hospital performance, (b) pressure of service recipients on prevention and effective and minimally invasive treatment methods improving the quality of life, (c) aging of the population and pressure to increase the effectiveness of medical care, (d) competence of medical professionals in the use of modern medical and non-medical technologies, (e) competence of managers of medical facilities in the field of implementing modern medical and non-medical technologies, (f) the share of the Polish healthcare sector of the medical tourism market.	Technological factors: (a) digitization, (b) telemedicine, (c) artificial intelligence, (d) personalized medicine, (e) development of new drug technologies, (f) development of new non-drug technologies, (g) The Agency for Health Technology Assessment and Tariff System technological support, (h) creation of a cooperating network in the field of HB-HTA.

The results of this part of the research were introduced in a report: *Analiza PEST dla wdrożenia HB-HTA w Polsce* (title in English: *PEST analysis for the implementation of HB-HTA in Poland)* prepared by M. Gałązka-Sobotka, I. Kowalska-Bobko, A. Frączkiewicz-Wronka, B. Więckowska, I. Skrzekowska-Baran, J. Gierczyński, A. Mela, K. Łach, K. Byszek, A. Dłutek, M. Furman (https://hbhta.pl).

#### Force Field analysis

This second stage of our research was identifying the determinants for implementing HB-HTA in Poland. The search for determinants of change—to prepare its effective implementation—is a frequently undertaken topic of research in the theory and practice of public management and public policies [49]. One of the tools of change management is the analysis of K. Lewin’s Force Field [50,51]. It requires the identification of factors that will potentially affect its implementation [52]. The idea behind Force Field analysis is that situations are maintained by the equilibrium between forces that drive change and others that resist change. For change to happen, the driving forces must be strengthened, or the restraining forces weakened. The application of Lewin’s [49–51,53–58] Force Field analysis led to identifying the forces driving and restraining the implementation of HB-HTA.

21 experts out of 50 who participated in the PEST analysis also participated in this stage of the study. This figure is due to the availability of experts and their willingness to continue the research. Their role was to identify driving and restraining forces applying force filed analysis on the 28 PEST factors. The face-to-face meeting (which took place at Lazarski University in Warsaw) was conducted by three moderators responsible for preparing the final report from this stage of research. The moderators were supported by two project coordinators, who were also performing the PEST analysis. The moderators were recognized by experts as known in the professional and scientific community. For the sake of scientific objectivity, the moderators did not provide experts with their opinions on the issues examined. Meetings were conducted according to a set scenario, and experts received a list of questions.

Assigning driving and restraining forces to each of the 28 PEST factors gave a total of 392 forces ((28 x7 + 28 x7 = 392)—PEST factors^x^driving forces + PEST factors^x^restraining forces)) behind the implementation of HB-HTA (the full list of identified forces is given in the supporting information, see S1 File). Following Force Field analysis methodology, the forces were then reduced by assigning them an appropriate number of points depending on their weight and importance. 140 driving and 140 restraining forces were identified, 5 driving and 5 restraining for each PEST factor respectively. This led to the identification of dominant forces and helped determine the chances of implementing HB-HTA.

At the first stage of the Force Field analysis, each of the separated factors is assigned 7 restraining forces and 7 driving forces. Next, it is necessary to prepare a balance of driving and restraining forces, which may appear during the implementation of a new solution in the organization or system. In the next step, each indicated driving and restraining force should be assigned a specific weight, i.e. the number of points reflecting the assessment of its impact. The ranking of the obtained results allows for the reduction of the forces to 5 driving and 5 restraining forces in relation to the indicated factor [59]. The assessment of the forces allows for the estimation of the chances of implementing the change and to identify those forces which are most important for the success of the change. It is also important to know which forces should be strengthened because they promote change, and which should be weakened because they inhibit it. The assessment of their impact on the course of change allows to design a rational strategy for its implementation [57].

The results of this part of the research were introduced in a report: *Analiza interesariuszy*, *sił hamujących i wspierających dla uwarunkowań politycznych*, *ekonomicznych*, *społecznych i technologicznych warunkujących proces wdrażania HB-HTA w polskim systemie ochrony zdrowia (title in English*: *Analysis of stakeholders*, *inhibiting and supporting forces for political*, *economic*, *social and technological determinants of HB-HTA implementation in the Polish healthcare system*), prepared by A. Frączkiewicz-Wronka, H. Kelm, K.Szymaniec-Mlicka (https://hbhta.pl).

## Results: Categorization of the main forces driving and restraining the implementation of HB-HTA (Gioia approach)

The results obtained in the PEST analysis and Force Field analysis led to the identification of the main forces driving and restraining the implementation of HB-HTA.

The next step in the study conducted by the team of researchers (all authors of this article) using the Gioia method was to combine similar factors with potential impact on the process of HB-HTA implementation in the Polish healthcare system into homogeneous thematic groups. This is a method of analyzing qualitative data which offers a “systematic approach to new concept development and grounded theory articulation” [60] and brings "qualitative rigor" and transparency to conducting and presenting inductive research.

In the presented study the Gioia approach is used to thematize and aggregate the identified factors conducive to the implementation of HB-HTA in the Polish healthcare system. Accordingly, codes were developed so that researchers could use them to organize and interpret data gathered in the research process.

The codes and coding process were adapted to the empirical material collected. Qualitative coding, used in grounded theory, follows a completely different logic than quantitative coding. In the latter, codes and categories exist before coding begins and are derived from the existing theory. In qualitative coding, codes “emerge from the context” because they are created during the coding process and emerge from this process following the synthesis of the descriptions prepared [27]. Coded descriptions were grouped into categories under the proposed names of phenomena. The analysis of relations between the most useful categories yielded the core category, which then became the axis of the created theory. The creation of categories requires that the level of abstraction is high enough to prevent creating a separate category for each event, while simultaneously low enough so that the proposed category actually reflects the core of the descriptions grouped around a given phenomenon [61]. At this stage, the categories that will develop into a medium-range theory are created.

The driving forces (140) and restraining forces (140) identified as a result of Force Field analysis were recognized as 1st order categories. Then they were grouped into 2nd order themes. The driving forces and restraining forces made up 19 thematic groups each. The final stage was to aggregate the 2nd order themes. In consequence, we created 6 aggregate dimensions for driving forces and 6 aggregate dimensions for restraining forces.

The following dimensions were identified as driving forces: (a) backed up, (b) capacity building, (c) environmental fit, (d) technological redefinition of services, (e) partnership development, and (f) relevance. Table 3 presents the grouping process of driving forces.

**Table 3:** Driving forces—Grouping process.

1st order categories	2nd order themes			Aggregate dimensions
Initial number of driving forces	Number of driving forces after 1^st^ grouping	Number of items in group	2^nd^ order group name	Final group name
196	140	5	Backed Up	Backed Up
		5	Trust	
		6	Consistency	
		12	Strategic And Operational Capacity	Capacity Building
		5	Leadership	
		5	Control Procedures	
		3	Risk Culture	
		5	Political Capacity	
		3	Decision Rationality	
		30	Adapting To the Challenges Of The National Environment	Environmental Fit
		5	Adaptation To the Challenges Of The International Environment	
		8	Economic Context	
		4	Resource Limitations	
		10	Evidence Base	Technological Redefinition Of Services
		16	New Technologies in Services	
		5	Networking	Partnership Development
		2	Partnership Development	
		5	Usefulness And Complementarity	Relevance
		6	Relevance	

The following dimensions were identified as restraining forces: (a) information asymmetry, (b) political ignorance, (c) economic constraints, (d) negligence, (e) unsound institutions, and (f) unsteady resources. Table 4 presents the grouping process of restraining forces.

**Table 4:** Restraining forces—Grouping process.

1st order categories	2nd order themes			Aggregate dimensions
Initial number of restraining forces	Number of restraining forces after 1^st^ grouping	Number of items in group	2^nd^ order group name	Final group name
196	140	9	Information Asymmetry	Information Asymmetry
		1	Politicization Of the Decision-Making Process	
		5	Political Sluggishness	Political Ignorance
		5	Marginalization	
		14	Political Ignorance	
		3	Incrementalism In Change	
		9	Political And Regulatory Inconsistency	
		4	Economic Cyclical Factors	Economic Constraints
		9	Budget Balance Constraint	
		9	Macroeconomical Speed	
		10	Healthcare Economical Speed	
		25	Negligence	Negligence
		5	Incompetence In Action	
		7	Institutional Hierarchy	Unsound Institutions
		3	Procedural Stiffening	
		4	Red Tape	
		7	Unsound Institutions	
		4	Unsteady Infrastructural Resources	Unsteady Resources
		7	Unsteady Human Resources	

**Fig 2:**
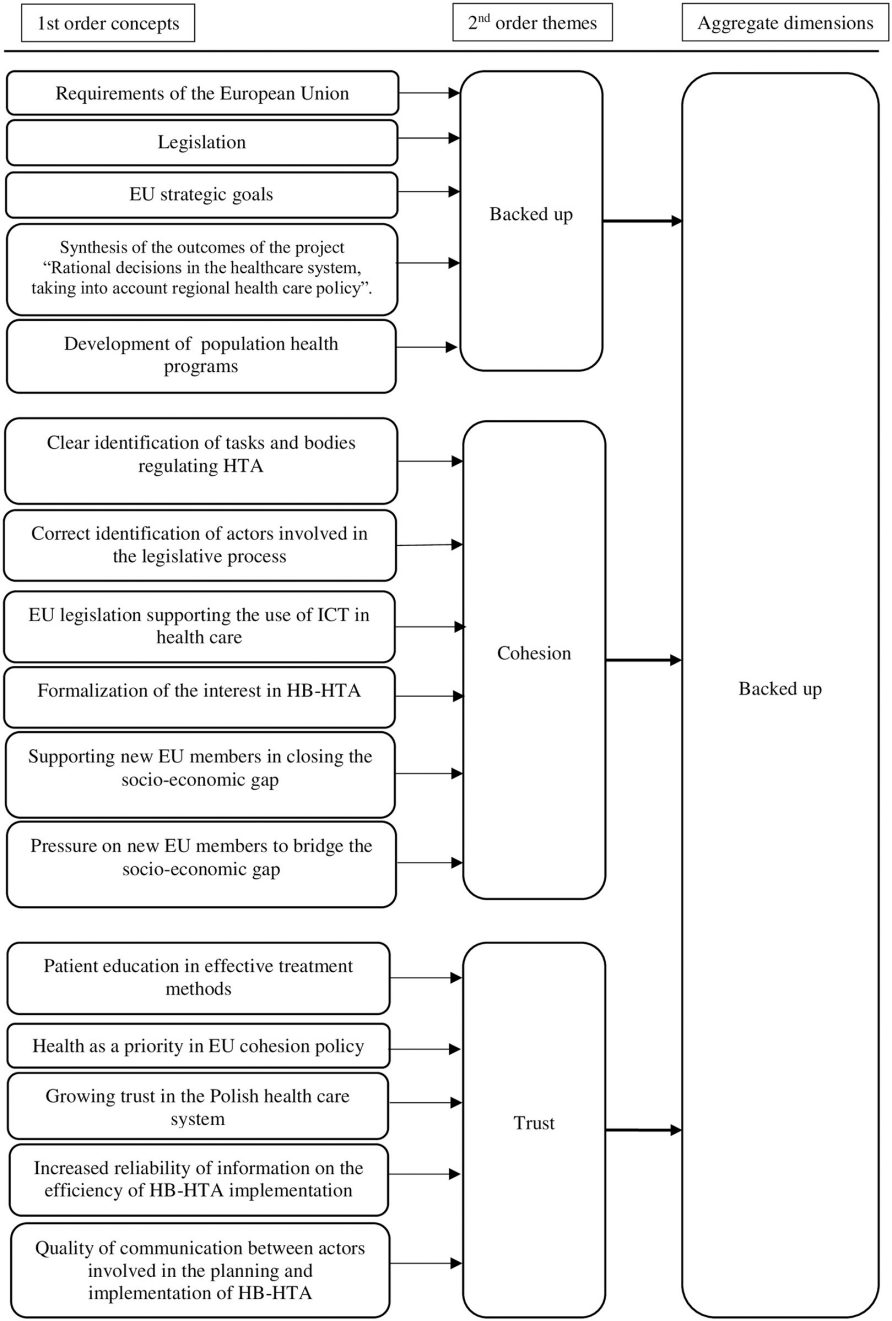
Sample of Gioia approach grouping process.

The figure below illustrates the grouping methodology, using the example of one selected—backed up—dimension within driving forces (see Fig 2).

## Discussion: Identified dimensions in the HB-HTA implementation

The process of introducing HB-HTA in Poland follows the assumptions of the pragmatic model of public responsiveness by combining social expectations with expert knowledge. The use of Force Field analysis for which we used the Gioia approach provides the knowledge necessary in the process of building and implementing a strategy, consisting of, among others, weakening the restraining forces and strengthening the forces driving the change.

The following quote confirms the great importance of analyzing the driving and restraining forces at work during the introduction of HB-HTA [62]:

“*Public administrators are and should be held accountable to a constellation of institutions and standards, including the public interest; statutory and constitutional law; other agencies; other levels of government; the media; professional standards; community values and standards; situational factors; democratic norms; and of course, citizens. Indeed, they are called upon to be responsive to all the competing norms, values and preferences of our complex governance system*”.

**Fig 3:**
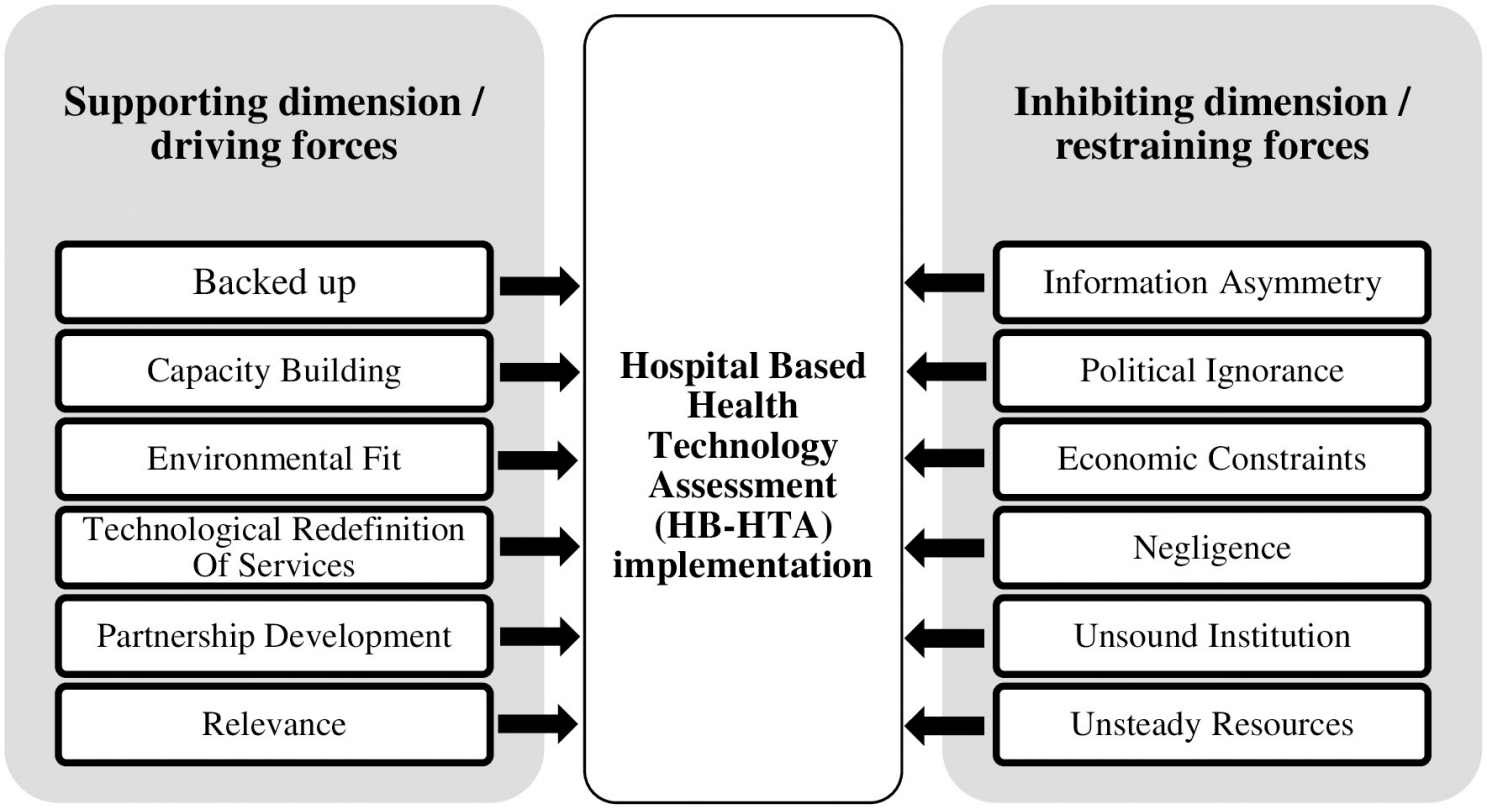
Model of dimensions supporting and inhibiting the HB-HTA implementation.

The grouping and interpretation process led to identifying the most important dimensions that can support or inhibit HB-HTA implementation in the Polish healthcare system (see Fig 3).

Below in the discussion we indicate their potential impact on the implementation of HB-HTA in the Polish healthcare system.

The following dimensions were identified as supporting: (a) backed up, (b) capacity building, (c) environmental fit, (d) technological redefinition of services, (e) partnership development, and (f) relevance.

**Backed Up**, defined as the “nature of the political contexts” [63], political backing [64], including legal and political frameworks, as well as informal institutions (i.e., norms, culture) that may enable or hinder individuals and organizations from carrying out their functions [65]. Backed Up gives implementing entities the assurance that their measures are not only regulated by law, but also supported by the guidelines and actions of transnational (the European Union), national (government and ministries, public universities, the Agency for Health Technology Assessment and Tariff System), regional (local government units) public institutions [66]. Backed Up is also the underlying certainty that an action is technically justifiable, supported by evidence, successful in other countries (or at a small scale domestically), and cost-effective [66].

In the context of HB-HTA, national legislation is becoming more important as a driving force, as in many areas it is increasing the decision-making autonomy of hospital managers, e.g., in terms of opportunities in telemedicine or digitization. The objectives adopted by the European Union with regard to healthcare systems, including the increased innovation, efficiency, and stability of healthcare systems, to a large extent facilitate the implementation of HB-HTA because, as a consequence of their adoption, a stream of financial resources has been initiated, e.g., for improving the competences of hospital managers or e-Health projects [67]. The process of change is also fostered by the public’s growing trust in the healthcare system, and because of which, the legitimacy of change is increasing among its stakeholders—organizations representing patients, trade unions, constituent bodies, hospitals, medical personnel, and patients.

According to Pichler et al., **Capacity Building** can be defined as “the process by which individuals and organizations develop or strengthen abilities related to understanding, providing input to, conducting, or utilizing HTA for health policy and decision making, as well as developing awareness and support in the environment within which HTA is being used” [65]. Capacity Building is not simply training; it goes beyond training individuals to include broader sets of changes in institutions, policies, and behaviors [68]. Potter and Brough indicate that systemic Capacity Building “improves diagnosis of sectoral shortcomings in specific locations, improves project/programme design and monitoring, and leads to more effective use of resources” [69]. They also point out the hierarchy of capacity building needs: (1) structures, systems, and roles, (2) staff and facilities, (3) skills, and (4) tools. In the Polish context, Capacity Building clearly supports the implementation of HB-HTA, especially in terms of operational and strategic competence development (experience in obtaining EU funds, development of skills relating to new technologies, development of managerial competences), leadership [70] (managers’ greater decision-making independence, flat structures and organizational learning), and the development of risk culture in organizations, which is an important element of introducing an innovation [71] such as HB-HTA. Hence, the development of risk management skills and risk culture based on the perception of risk not only in terms of costs, but above all, benefits, supports the implementation of HB-HTA.

**Environment Fit** [72]–“Policy is not a vacuum; it is the result of the interaction of all background factors with the desires and decisions of those who make policies” [73]. Accordingly, Environmental Fit means that actions need to constitute an adequate response to challenges in the political, social, and economic environment [74,75]. The environmental factors that have the strongest impact on the process of implementing HB-HTA in Polish hospitals include social expectations that public services will be of higher quality, which “force” managers to seek opportunities to streamline processes in the organization. A strong factor facilitating change is also the ageing society, which creates the need to develop new areas of health care services that would meet emerging health challenges, including the development of health care services based on telemedicine, which will improve the availability of health care services for people with limited mobility. A potential factor driving the implementation of HB-HTA is also the shortage of medical personnel, which gives an impulse to seek new forms of health care service provision and assign new roles to current medical professions [76].

**Technical Redefinition of Services** means considerable changes in how health care services are provided, which are the result of accelerated technological development. Digitization, development of artificial intelligence, ICT, and personalized medicine create new opportunities for managers to grow their organization by improving the quality of services, but at the same time, they require “unlearning” the old patterns of thinking and acting [77], as well as improving the decision-making process, which involves the implementation of HB-HTA. Seeing a great variety of new technologies, managers will seek out and adapt tools (including HB-HTA) that will allow them to comprehensively assess individual solutions and their potential impact on the organization’s operations.

**Partnership Development** involves joint actions and cooperation with entities working with HB-HTA. Partnership Development as a driving force operates in two areas [5]: (1) cooperation with regional, national, or European entities responsible for health technology assessment and (2) establishing partnerships between hospitals implementing HB-HTA. Partnership Development drives the implementation of HB-HTA by enabling resource sharing, mainly knowledge and information, and mutual learning and teaching of HTA by hospitals. This is also facilitated by the formalization of collaboration networks, networking, and the use of networks as a change strategy in the healthcare system [78].

**Relevance** refers to the extent to which the proposed changes meet the needs/problems/expectations of stakeholders [79]. A stakeholder is an individual or group that may affect one’s ability to achieve the objectives of the organization, or which may be affected by the organization if it reaches its goals [80]. In terms of health technology assessment, Street et al. identify the following stakeholders: patients, consumers, carers, industry representatives, healthcare providers, employers, health insurers, and other payers. They also indicate that “*members of the public and taxpayers may also be considered to be distal stakeholders in the HTA process because of their interest in a viable effective health system and the judicious use of public funds”* [81]. Relevance is one of the evaluation criteria for public interventions [82]. With regard to the implementation of HB-HTA, it is particularly important to develop appropriate (legal, financial) incentive instruments for hospitals so that they increase interest in and commitment to the process of change. The incentive system should be based on material stimuli. Other strong change drivers are the rationality and functionality of the solution in terms of positioning HB-HTA in the existing healthcare system, as they show how the change will benefit the stakeholders.

The following dimensions were identified as inhibiting: (a) information asymmetry, (b) political ignorance, (c) economic constraints, (d) negligence, (e) unsound institutions, and (f) unsteady resources.

**Information Asymmetry** [83] is an inherent feature of the system, characterizing the relations between patient-doctor, doctor-therapeutic entity, and therapeutic entity-governmental/self-government institution/paymaster [84]. Information Asymmetry is a lack of information awareness. It exists when a party or parties possess greater informational awareness in a given situation relative to other participating parties [85]. Information Asymmetry restrains the process of implementing HB-HTA mainly because no clear guidelines on the procedure for using HB-HTA are provided to hospitals by the implementing institutions. Strong asymmetry exists in the area of medical professionals’ knowledge about new health technologies—drug and non-drug related. Another important problem involves the relatively low tendency of hospitals to disseminate knowledge about treatment results and costs. Information Asymmetry also makes it difficult for hospital managers to access the knowledge necessary in HB-HTA to analyze new health technologies in terms of the entity’s strategic goals [86].

**Political Ignorance** can be defined as the lack of knowledge of political decision-makers, knowledge suppression and limitation [87], or intentional use of ignorance (strategic ignorance) [88]. Political Ignorance, understood in this way, can be treated as a source of action related to the agency of public policy actors, where ignorance consists of *“any actions which mobilize*, *manufacture*, *or exploit unknowns in a wider environment”* [89]. In relation to the implementation of HB-HTA, the restraining nature of Political Ignorance mainly involves a lack of understanding or residual understanding of ongoing processes and political sluggishness referred to as the politics of time [88,90], i.e., the deliberate use of time/schedules to delay the implementation of HB-HTA through, for example, the absence of administrative procedures. The process of changes in Polish hospitals is also negatively affected by the lack of solutions adopted to promote the implementation of HB-HTA units into the hospital structure, failure to take into account the emerging population needs in the vision of the implementation of HB-HTA, the incremental and political preparation of the implementation of HB-HTA missing relevant arguments, the failure to assess or the unreliable assessment of the effects of the proposed (central/regional) HB-HTA model, lack of a transparent and clear vision of the organizational structure supporting the implementation of HB-HTA, failure to take into account the results presented in audit documents, the lack of review of an organization’s own activity, and the undervalued results of the work paramedical specialists performed (e.g., public health graduates) by the health care sector.

**Economic Constraints** refer to the general macroeconomic conditions of the country and factors specific to the health care sector. It is a significant restraining force because the introduction of changes into public policy is always performed within the framework of specific economic determinants [91,92]. Among macroeconomic determinants, the process of change is adversely affected by the need to maintain a balanced budget [93] and, consequently, introduce budgetary constraints, including those on health care spending. The process of change may also be hindered by economic factors [94] an economic slowdown or downturn translate into lower health insurance premiums and sets back the implementation of HB-HTA. Apart from general macroeconomic factors, the process of implementing HB-HTA is also hampered by economic forces within the healthcare system—macroeconomic and health care economic speed [90], which should be understood as the failure of the system to keep up with changes in economic conditions occurring at a rapid pace [67]. The factors negatively impacting the process of change include diminishing resources coming from EU funds, the necessity to recruit new staff as well as modernizing and maintaining infrastructure, insufficient funds for financing scientific research, and the poor financial situation of hospitals, which hinders access to modern health technologies.

**Negligence**, defined as a deliberate, conscious, and planned decision made by implementing entities to discontinue certain actions, includes resigning from the intended or unintended outcome of an action. Regarding Negligence, it is important to be able to exert influence on actions, for example, by changing priorities, exhibiting disinvolvement, or showing weaker commitment than expected [66]. The restraining effect of Negligence may ultimately lead to failure [95]. Actions restraining the implementation of HB-HTA in the area of Negligence are a result of, for example, the attitude of medical personnel toward the process of change, the lack of knowledge and competences of hospital management and their reluctance to gain this knowledge and develop new competences, as well as weaknesses in the decision-making process due to, among other things, a lack of support for the process.

The **Unsound Institution** refers to the lack of readiness to implement change on the side of the implementing entity/system. Lack of readiness translates into the inability to act effectively in areas such as planning, coordination, resource allocation, quality control, monitoring and joint implementation [66]. The Unsound Institution in the process of implementing HB-HTA draws on old management models, including institutional hierarchy, procedural stiffening, and red tape. Excessive hierarchy and imbalance in relations between the parties involved in the process of implementing HB-HTA, especially paired with overall reluctance to create regional inter-institutional networks responding to concerns about limited competences and costs of participation in the network, hinder the implementation of HB-HTA in Polish hospitals. Red tape can be defined as *“rules*, *regulations*, *and procedures that remain in force and entail a compliance burden for the organization but have no efficacy for the rules’ functional object or organization”* [96]. Prolixity, overregulation, complex procedures and the pursuit of excessive compliance in the Polish healthcare system are important forces restraining the process of implementing HB-HTA by limiting the discretionary power of the entities involved in the process of change, which is related to the concept of stakeholder red tape, defined as *“a rule that remains in force and entails a compliance burden but serves no objective valued by a given stakeholder group”* [97].

**Unsteady Resources** means that the entities responsible for solving a problem or implementing an action lack the required resources. Additionally, the formulation of particular solutions does not involve the actual allocation of new resources; thus, planning, implementation, and stability are affected and, as a result, so is the effectiveness of the implemented projects [66,98]. In the area of infrastructural resources, the greatest barrier to the implementation of HB-HTA is insufficient IT infrastructure and the related delays in digitization of health care, which hinder access to information necessary for the proper assessment of health technologies. In the area of human resources, an important restraining force is the drainage of the labor market for medical professions and, consequently, staff shortages among doctors and nurses (emigration), and in groups of workers supporting treatment processes (coordinators, medical secretaries). The implementation of HB-HTA in the practice of Polish hospitals is also hampered by the lack of highly qualified management staff in hospitals.

## Conclusions

The aim of our study was to identify and analyze forces which will shape a dynamic process in determining the implementation of HB-HTA. The main value of the research is found in the opinions of experts who participated in the focus study and played the role of "voices from the field" [99]. Their expert knowledge constituted a valuable database, which was then analyzed using various research methods [100]. Our study reflects the interpretive research tradition [27] in qualitative research, where the essence of phenomena is constructed by individuals taking part in the processes studied. The authors of the study decided to take an interpretative approach to research because it offers certain desired benefits [101]. The complex research process used has been carefully developed to allow for the most accurate and multi-faceted analysis of the problem under investigation. This enabled identification of the differences in the attitudes and opinions of the experts participating in the study. Although qualitative research generally does not allow statistically significant conclusions to be drawn, it allows analysis of socially relevant issues, such as the conditions for implementing HB-HTA. The qualitative research allowed for exploration of driving and restraining forces in the implementation of HB-HTA as well as understanding and assessing the conditions in which complex interventions can be introduced and standardized in healthcare. The authors of this paper are aware of the fact that conducting qualitative research is difficult and faces many limitations. In the literature, the most important factors for obtaining valuable results of the analysis conducted using Force Field analysis emphasize the importance of the involvement and quality of knowledge of experts participating in this type of research, meeting facilitators, as well as analysts processing the obtained material. Their figural and semantic knowledge, as opposed to procedural knowledge, about design depends more on information based on "dense and rich" first-hand descriptions, which no amount of empirical knowledge obtained in experimental studies can provide [102,103]. As indicated above Force Field analysis—in order to provide reliable material for building a strategy for implementing change in a public system—requires full involvement of the people involved and knowledge of the realities of the area to which it is dedicated. With a lack of in-depth knowledge and full involvement of experts, facilitators and analysts in the research process will not be able to prepare a realistic picture of supporting and opposing forces. Another threat to the potential results may be the inability of the moderators to gain consensus among the participants [104] of the expert panels and thus obtain poor or unreliable material as a basis for analytical work. These statements are confirmed in numerous scientific articles [105]. This is particularly evident in the analyses conducted in relation to the implementation of changes in public systems, for example, health care, education, culture, and others [106]. In the indicated systems, whose activities are publicly funded, political interest and often conflicting interests of stakeholders contribute to the formulation of conclusions not fully based on rational grounds [107]. In order to design future implementation activities well, a thorough understanding of the current state is necessary, which in our opinion was ensured by the selection of the people participating in each stage of the implemented study. The obtained material contributed to a better understanding of both opportunities and threats to the introduction of HB-HTA solution to the Polish healthcare system. This is important for patients, clinicians, healthcare managers, and decision makers. It is also important for researchers from various fields who attempt to carry out and evaluate such changes [108]. Using study typology of Fisher and Hamer, conducted research could be included in policy and program environment study type [109] typical for the governance model of Public Management.

The concept of governance, which is assumed to be separate and going further than New Public Management, starts from the humanist rationale of understanding public management and refers to the responsible co-creation of reality by the citizens, with the servant role of administration and public authorities. In practice, this means co-determination, cooperation, and the use of democratic procedures in deciding on the hierarchy and the way to meet the needs of society.

Kooiman defines governance as a pattern of conduct or structure that emerges in a socio-political system as a common result or consequence of the interactive intervention efforts of all its active participants [110]. This understanding of governance means that there is a need to continuously revive/build the relationship between society and its constituent citizens and governing decision-makers, i.e., responsiveness.

The latter is a consequence of extending the responsibility for meeting public needs to many organizations operating in network systems, for which improving the quality of life of citizens is the primary goal [111].

An example can be, on the one hand, organizations operating in the healthcare system and, on the other hand, entities creating healthcare policy objectives, the implementation of which is to contribute to the improvement of its state among citizens. Due to the fundamental right of economics to unlimited needs and limited resources, it is important to choose such objectives and their subsequent implementation which will be socially just and economically efficient.

It should be remembered that responsiveness does not consist in unconditionally meeting the requirements and desires of society, but in a highly individual approach consisting of the selection of appropriate mechanisms of public participation based, among others, on subjective criteria. Moreover, acting in accordance with this concept, it should be remembered that there are no universal "answers", and that every issue or problem requires rethinking and applying creative, progressive solutions. In political science, responsiveness refers to the relationship between the dreams, demands and tastes of citizens and the decisions that directly or indirectly affect them, as well as the ability of politicians to select responses that respond to current preferences and moods of members of society. On the grounds of public management, however, responsiveness is the ability of rulers to diagnose and verify subsequent problems and to solve them effectively. It is necessary in this process to apply tools adequate to the problem and to conduct the research procedure carefully so that the results obtained have an application value. The assumptions indicated were fulfilled in the research, the course and results of which are discussed in this article.

In practice, the above considerations lead to the conclusion that it is necessary in the process of creating an agenda for implementation HB-HTA to focus not only on maintaining the original predominance of forces, but also on weakening the restraining forces, e.g., economic constrain, where the main problem is the lack of financial resources. It is necessary not only to provide a larger pool of funds within the whole system, but also to introduce a system of financial incentives for entities actively using HB-HTA in their activities and, as a result, achieving greater efficiency of operation. Entities opting for HB-HTA must not only have free financial resources to enable them to invest, but they must also have the means to operate HB-HTA units within their structures. As Poder [112] points out, one of the most important reasons for the lack of implementation of decisions resulting from HB-HTA reports is precisely financial limitations.

Among the forces supporting the process of change, the most important is the Backed Up, especially the inspiration to introduce the change, which materializes in the project "Implementation of the Hospital-Based HTA (HB-HTA) system—Hospital Assessment of Innovative Medical Technologies". Not only does it comprehensively analyze the possibilities of HB-HTA implementation in the Polish healthcare system, but, above all, it is an important beginning of the discussion among managers of medical entities, becoming a kind of inspiration to look for possibilities of improving the decision-making process at the hospital level.

For the process of the implementation and further development of HB-HTA, it is crucial to continuously strengthen the autonomy of hospitals and to create as much decision-making space as possible, especially in conditions of continuous technological progress. Intense technological progress even forces the implementation of HB-HTA to make it easier for managers to navigate through the maze of new drug and non-drug technologies, especially under conditions of quite limited resources.

The research carried out clearly indicated the importance of the human factor in the implementation process, both as a supporting force and, above all, in terms of inhibiting forces. Staff shortages and insufficient management competencies may, if not addressed, strengthen the scale of their impact, especially in the crucial phase of HB-HTA implementation already in a particular organization. Thus, it is advisable to arouse the need for change among managers with simultaneous training activities in the HB-HTA methodology [30].

To sum up, we emphasis that this study, is the first time—that the authors are aware of—that the determinants of the implementation of HB-HTA in Poland, as a complex and dynamic social problem, were analyzed. The obtained results of the study contributed to the choice of HB- HTA implementation in selected hospitals in Poland. The pilot implementation of HB- HTA started in Poland in June 2021 (www.hbhta.pl).

## Supporting information

S1 File Matrix of identified forces. The full list of identified driving and restraining forces.
(PDF)

## Abbreviations

AdHopHTA: Adopting Hospital Based Health Technology Assessment
AOTMiT: Agency of Health Technology Assessment and Tariff System
CEDIT: Le Comité d´Evaluation et de Diffusion des Innovations Technologiques
CRD database: The Centre for Reviews and Dissemination database
EIP-AHA: Council of the European Union and the European Innovation Partnership on Active and Healthy Aging
EMBASE: Excerpta Medica dataBASE
EUNetHTA: European Network for Health Technology Assessment
GENESIS: Grupo de Evaluación de Novedades, Estandarización e Investigación en Selección de Medicamentos
GOSPOSTRATEG: Strategic program of scientific research and development
HB-HTA: Hospital Based Health Technology Assessment
HiT: European Observatory on Health Systems, Healthcare Systems in Transition
HTA: Health Technology Assessment
HTAi: Health Technology Assessment International
INAHTA: International Network of Agencies for Health Technology Assessment
ISI Web of Knowledge: Institute of Scientific Information
ISPOR: International Society for Pharmacoeconomics and Outcomes Research
NHF: National Health Fund
PEST: Political, Economic, Social and Technological Analysis
PubMed (Medline): Public Medline
WHO database: World Health Organization database

## References

[1] Osborne SP, Brown K. Managing change and innovation in public service organizations London and New York: Routledge; 2012.

[2] Kanter RM. Ten Reasons People Resist Change. Harvard Business Review. 2012 Sep.

[3] INHATA. www.inahta.org. [Online].; 2020 [cited 2020 04 30.

[4] EUnetHTA. www.eunethta.eu. [Online].; 2020 [cited 2020 4 30.

[5] Sampietro-Colom L, Lach K, Cicchetti A, Kidholm K, Pasternack I, et al. The AdHopHTA handbook: a handbook of hospital based Health Technology Assessment. [Online].; 2015 [cited 2020 4 7. http://www.adhophta.eu/sites/files/adhophta/media/adhophta_handbook_website.pdf.

[6] Liao Y. Municipal Managers’ Responsiveness to Public Demands: Connecting Attitudinal Willingness, Behavioral Willingness, Environmental and Organizational Factors.; 2013 [cited 2020 4 10. works.bepress.com/yuguo-liao/8.

[7] Vigoda E. From Responsiveness to Collaboration: Governance, Citizens, and the Next Generation of Public Administration. Public Administration Review. 2002 Sep.

[8] Ostrom E. The design of institutional arrangements and the responsiveness of the police. In Rieselbach L, editor. People vs. government.: Bloomington: Indiana University Press.; 1975. p. 274–299.

[9] Forouzan S, Padyab M, Rafiey H, Ghazinour MD, et al. Measuring the Mental Health-Care System Responsiveness: Results of an Outpatient Survey in Tehran. Frontiers in Public Health. 2016 Mar. doi: 10.3389/fpubh.2015.00285 26858944 PMC4728407

[10] Chao J, Lu B, Zhang H, et al. Healthcare system responsiveness in Jiangsu Province, China. BMC Health Service Research. 2017;(31). doi: 10.1186/s12913-017-1980-2 28086950 PMC5237227

[11] Daneshkohan A, Zarei E, Ahmadi-Kashkoli S. Health system responsiveness: A comparison between public and private hospitals in Iran. International Journal of Healthcare Management. 2018.

[12] Hamid SA, Begum A. Responsiveness of the urban primary health care delivery system in Bangladesh: A comparative analysis. International Journal of Health Planning and Management. 2019; 34(1). doi: 10.1002/hpm.2626 30146682

[13] Fried R. Performance in American bureaucracy Boston: Little Brown; 1976.

[14] Bryer T. Toward a relevant agenda for a responsive public administration. Journal of Public Administration Research and Theory. 2007; 17(3): p. 479–500.

[15] Rourke FE. Responsiveness and neutral competence in American bureaucracy. Public Administration Review. 1992; 52(6): p. 539–546.

[16] Saltzstein GH. Bureaucratic responsiveness: Conceptual issues and current research. Journal of Public Administration and Theory. 1992; 2(1): p. 63–88.

[17] Stivers C. The listening bureaucrat: Responsiveness in public administration. Public Administration Review. 1994; 54(4): p. 364–369.

[18] Schumaker P. Policy responsiveness to protest-group demands. Journal of Politics. 1975; 37(2): p. 488–521.

[19] Rourke FE. Bureaucracy, politics, and public policy Boston: Little Brown; 1969.

[20] Yang K, Pandey SK. Public responsiveness of government organizations: testing a preliminary model. Public Performance and Management Review. 2007; 31(2): p. 215–240.

[21] Demirdjian G. A 10-year hospital-based health technology assessment program in a public hospital in Argentina. International Journal of Technology Assessment in Health Care. 2015; 31(1–2): p. 103–110. doi: 10.1017/S0266462315000124 25952708

[22] Bellah R, Madsen R, Sullivan W, Swide A, Tipton S. The Good Society New York: Knopf; 1991.

[23] Yang K. Responsiveness in network governance: Revisiting a fundamental concept: Symposium Introduction. Public Performance and Management Review. 2007; 31(2): p. 131–143.

[24] Shaffer WR, Weber RE. Policy responsiveness in the American States Beverly Hills, CA: Sage; 1974.

[25] Kowalska-Bobko I. Decentralizacja a systemy zdrowotne. W poszukiwaniu rozwiązań sprzyjających zdrowiu Cracow: Jagiellonian University; 2017.

[26] Gałązka-Sobotka M, Kowalska-Bobko I, Lach K, Mela A, Furman M, Lipska I. Recommendations for the Implementation of Hospital Based HTA in Poland: Lessons Learned From International Experience. Front. Pharmacol. 2021;(11). doi: 10.3389/fphar.2020.594644 34054508 PMC8155722

[27] Gephart RP. Qualitative research and the academy of management journal. Academy of Management Journal. 2004; 47(4): p. 454–462.

[28] Glaser BG, Strauss AL. Basic of Grounded Theory Analysis. Emergence vs Forcing Mill Valley, CA: Sociology Press; 1992.

[29] Glaser BG, Strauss AL. Doing Grounded Theory. Issues and Discussions Mill Valley, CA: Sociology Press; 1998.

[30] Mills J, Bonner A, Francis K. The development of constructivist grounded theory. Int. J. Qualitative Methods. 2006; 5(1).

[31] Glaser BG, Strauss AL. Odkrywanie teorii ugruntowanej. Strategie badania jakościowego Cracow: Nomos; 2009.

[32] Blumer H. Symbolic interactionism: perspective and method Berkeley: University of California Press; 1969.

[33] Strauss A, Corbin J. Basics of Qualitative Research: Grounded Theory Procedures and Techniques Newbury Park, CA: Sage; 1998.

[34] Patton MQ. Qualitative Research and Evaluation Methods. 3rd ed. Thousand Oaks, CA: Sage Publications Inc.; 2002.

[35] Charmaz K. Constructing Grounded Theory: A Practical Guide through Qualitative Research London: Sage Publications Ltd.; 2006.

[36] Czarniawska-Joerges B. Exploring Complex Organizations. A Cultural Perspective Newbury Park: Sage; 1992.

[37] Czarniawska-Joerges B. Wind of organizational change. How ideas translate into objects and actions. In Bacharach S. G P M B, editor. Research in the sociology of organizations. Greenwich, CT: JAI Press; 1995.

[38] Czarniawska B. Trochę inna teoria organizacji. Organizowanie jako konstrukcja sieci działań Warsaw: Poltext; 2010.

[39] Denzin N. Sociological methods. A Sourcebook New Brunswick: Transaction Publishers; 2006.

[40] Parra-Luna F. A Model for Measuring the Performance of Social Systems. In PL F., editor. The Performance of Social Systems. Perspectives and Problems. New York/Boston/Dordrecht/London/Moscow: Kluwer Academic/Plenum Publishers; 2000.

[41] Glaser BG, Strauss AL. Doing Grounded Theory. Issues and Discussions Mill Valley, CA: Sociology Press; 1998.

[42] Geertz C. The Interpretation of Cultures: Basic Book; 2017.

[43] Rorty R. Consequences of pragmatism Minneapolis, MN: University of Minnesota Press; 1982.

[44] Suddaby R. From the Editors. What Grounded Theory is Not. Academy of Management Journal. 2006; 49(4): p. 633–642.

[45] Sammut‐Bonnici T, Galea TD. PEST analysis. In Wiley Encyclopedia of Management, Volume 12. Strategic Management.: Wiley; 2015.

[46] Kim-Keung Ho J. Formulation of a Systemic PEST Analysis for Strategic Analysis. European Academic Research. 2004 August; 2(5).

[47] Kruger R, Casey M. Focus Groups: A Practical Guide for Applied Research. 3rd ed. CA: Thousand Oaks, Sage Publications; 2000.

[48] Cooper P, Diamond I, Gould C, Partridge J. Choosing and Using Contraceptives: Consumer Experiences in Wessex. Report to the Wessex Regional Health Authority Measures in Family Planning Steering Group. University of Southampton, Department of Social Statistics; 1992.

[49] Burnes B. Kurt Lewin and the Planned Approach to Change: A Re-appraisal. Journal of Management Studies. 2004; 41(6): p. 977–1002.

[50] Lewin K. Field theory in social science New York: Harper; 1951.

[51] Burnes B. Kurt Lewin (1890–1947): The Practical Theorist. In DBSe al., editor. The Palgrave Handbook of Organizational Change Thinkers.: Palgrave Macmillan; 2017.

[52] Efe AJ. Change Management Strategies in Policies and Reforms and Administrative Functions Competence in Delta State Colleges of Education. World Journal of Education. 2018; 8(4): p. 188–197.

[53] Lewin K. Field theory and learning. In D C, editor. Field Theory in Social Science: Selected Theoretical Papers by Kurt Lewin. London: Social Science Paperbacks; 1952. p. 60–86.

[54] Lewin K. Constructs in field theory. In D C, editor. Field Theory in Social Science: Selected Theoretical Papers by Kurt Lewin. London: Social Science Paperbacks; 1952. p. 30–42.

[55] Schein EH. Kurt Lewin’s change theory in the field and in the classroom: Notes toward a model of managed learning. Systems practice. 1996; 9(1): p. 27–47.

[56] Lunenburg FC. Forces for and Resistance to Organizational Change. National Forum of Educational Administration And Supervision Journal. 2010; 27(4).

[57] Burnes B, Cooke B. Kurt Lewin’s Field Theory: A Review and Re-evaluation. International Journal of Management Reviews. 2013; 15: p. 408–425.

[58] Sarayreh BH, Khudair H, Barakat E. Comparative study: the Kurt Lewin of change management. International Journal of Computer and Information Technology. 2013; 2(4): p. 626–629.

[59] Lehmann S. Bridging Strategies and Action: Towards a Method for Change Management in Danish Emergency Management Organizations. Journal of Change Management. 2017; 17(2): p. 138–154.

[60] Gioia DA, Corley KG, Hamilton AL. Seeking Qualitative Rigor in Inductive Research: Notes on the Gioia Methodology. Organizational Research Methods. 2012; 16(1): p. 15–31.

[61] Martin P, Turner BA. Grounded Theory and Organizational Research. The Journal of Applied Behavioral Science. 1986; 22(2): p. 141–157.

[62] Denhardt J, Denhardt R. The New Public Service: Serving, not steering: Routledge; 2016.

[63] Shiffman J, Smith S. Generation of political priority for global health initiatives: a framework and case study of maternal mortality. Lancet. 2007; 370: p. 1370–79. doi: 10.1016/S0140-6736(07)61579-7 17933652

[64] Hill R, Gonzalez W, Pelletier DL. The Formulation of Consensus on Nutrition Policy: Policy Actors’ Perspectives on Good Process. Food and Nutrition Bulletin. 2011; 32(2): p. 92–104. doi: 10.1177/15648265110322S206 21916118

[65] Pichler F, Oortwijn W, Ruether A, Trowman R. Defining capacity building in the context of HTA: a proposal by the HTAi Scientific Development and Capacity Building Committee. International Journal of Technology Assessment in Health Care. 2019;: p. 1–5. doi: 10.1017/S0266462319000631 31506132

[66] Escobar-Alegria JE, Frongillo EA, Blake CE. Sustainability of food and nutrition security policy during presidential transitions. Food Policy. 2019; 83: p. 195–203. 10.1093/cdn/nzaa161 PMC7792567 33447696

[67] Holecki T, Kowalska-Bobko I, Fraczkiewicz-Wronka A, Wegrzyn M. Realization of the EU’s Cohesion Policy in Health Care in the Visegrad Group Countries in the Perspective 2014–2020. Front. Public Health. 2020; 8(133). doi: 10.3389/fpubh.2020.00133 32391306 PMC7191030

[68] Bryce J, Coitinho D, Darnton-Hill I, Pelletier D, Pinstrup-Andersen P. Maternal and child undernutrition: effective action at national level. Lancet. 2008; 371: p. 510–26. doi: 10.1016/S0140-6736(07)61694-8 18206224

[69] Potter C, Brough R. Systemic capacity building: a hierarchy of needs. Health Policy And Planning. 2004; 19(5): p. 336–345. doi: 10.1093/heapol/czh038 15310668

[70] Szymaniec-Mlicka K. Resource-based view in strategic management of public organizations–a review of the literature. Management. 2014; 18(2): p. 19–30.

[71] Brown L, Osborne SP. Risk and Innovation. Public Management Review. 2013; 15(2): p. 186–208.

[72] Miller D. Environmental Fit Versus Internal Fit. Organization Science. 1992; 3(2): p. 159–178.

[73] Guy Peters B. American Public Policy: Promise and Performance: Sage; 2019.

[74] Birkland TA. An Introduction to the Policy Process Theories, Concepts, and Models of Public Policy Making. 3rd ed. London and New York: Routledge Taylor & Francis Group; 2015.

[75] Kelm H. Skuteczność polityki rodzinnej w warunkach regresu demograficznego w Polsce (The effectiveness of family policy in the context of demographic regression in Poland) Cracow: Cracow University of Economics; 2018.

[76] Kowalska-Bobko I, Gałązka-Sobotka M, Frączkiewicz-Wronka A, Badora-Musiał K, Buchelt B. Skill mix in medical and about medical professions. Medycyna Pracy. 2020; 71(3). doi: 10.13075/mp.5893.00817 32118874

[77] Stenvall J, Kinder T, Kuoppakangas P. Unlearning and public services–A case study with a Vygotskian approach. Journal of Adult and Continuing Education. 2018; 24(2): p. 188–207.

[78] Weible CM, Heikkila T, deLeon P, Sabatier PA. Understanding and influencing the policy process. Policy Sciences. 2012; 45: p. 1–21.

[79] deLeon P, Steelman TA. Making Public Policy Programs Effective and Relevant: The Role of the Policy Sciences. Journal of Policy Analysis and Management. 2001; 20(1): p. 163–171.

[80] Frączkiewicz-Wronka A, Szymaniec-Mlicka K, Dyaczyńska K, KotowskI P. The impact and importance of stakeholders of the health units to the managerial decision-making process. In Rudawska I, editor. Challenges for healthcare reform in Europe. Szczecin; 2011.

[81] Street J, Stafinski T, Lopes E, Menon D. Defining the role of the public in Health Technology Assessment (HTA) and HTA-informed decision-making processes. International Journal of Technology Assessment in Health Care. 2020;: p. 1–9. 10.1017/S0266462320000094 32151290

[82] European Commission. EVALSED: The resource for the evaluation of Socio-Economic Development. [Online].; 2013 [cited 2020 4 6. https://ec.europa.eu/regional_policy/en/information/publications/evaluations-guidance-documents/2013/evalsed-the-resource-for-the-evaluation-of-socio-economic-development-evaluation-guide.

[83] de Vries EF, Drewes HW, Struijs JN, Heijink R, Baan CA. Barriers to payment reform: Experiences from nine Dutch population health management sites. Health Policy. 2019; 123: p. 1100–1107. doi: 10.1016/j.healthpol.2019.09.006 31578167

[84] Major I. Two-Sided Information Asymmetry in the Healthcare Industry. International Advances in Economic Research. 2019; 25: p. 177–193.

[85] Clarkson G, Jacobsen TE, Batcheller AL. Information asymmetry and information sharing. Government Information Quarterly. 2007; 24: p. 827–839.

[86] Kidholm K, Ølholm M, Birk-Olsen AM, Cicchetti A, et al. Hospital managers’ need for information in decision-making–An interview study in nine European countries. Health Policy. 2015; 119(11): p. 1424–1432. doi: 10.1016/j.healthpol.2015.08.011 26362086

[87] Roberts J. Organizational Ignorance: Towards a Managerial Perspective on the Unknown. Management Learning. 2012; 44(3).

[88] Chailleux S. Strategic ignorance and politics of time: how expert knowledge framed shale gas policies. Critical Policy Studies. 2019.

[89] McGoey L. The Unknowers: How Strategic Ignorance Rules the World: Zed Books Ltd.; 2019.

[90] Santiso J. Political sluggishness and economic speed: a Latin American perspective. Social Science Information. 2000; 39(2): p. 233–253.

[91] Tendera-Wlaszczuk H, Kamarad E, Kelm H, Natanek M. The impact of the implemented welfare state concept on leveling the socio-economic distance of the European Union new Member States to the EU-15. In European Union Ten Years after the Lisbon Treaty. Warsaw: Difin; 2020.

[92] Kelm H. Welfare states’ reforming in the post-crisis European Union. In Brańka T, Skrzypczyńska J, editors. Getting Europe back to work. Crisis (re)production and crisis overcoming in Europe. Poznan: Adam Mickiewicz University; 2019.

[93] Moran M, Rein M, Goodin RE, editors. The Oxford Handbook of Public Policy: Oxford University Press; 2006.

[94] Birkland TA. An Introduction to the Policy Process Theories, Concepts, and Models of Public Policy Making. 3rd ed. London and New York: Routledge Taylor & Francis Group; 2015.

[95] McConnell A. A public policy approach to understanding the nature and causes of foreign policy failure. Journal of European Public Policy. 2016. doi: 10.1080/13501763.2015.1085073 28018131 PMC5175581

[96] Kaufmann W, Taggart G, Bozeman B. Administrative Delay, Red Tape, and Organizational Performance. Public Performance & Management Review. 2019; 42(3): p. 529–553.

[97] Bozeman B, Anderson DM. Public Policy and the Origins of Bureaucratic Red Tape: Implications of the Stanford Yacht Scandal. Administration & Society. 2014;: p. 1–24.

[98] Mur-Veeman I, van Raak A, Paulus A. Comparing integrated care policy in Europe: Does policy matter? Health Policy. 2008; 85: p. 172–183. doi: 10.1016/j.healthpol.2007.07.008 17767975

[99] van Eyk H, Baum F. Evaluating Health System Change-Using Focus Groups and a Developing Discussion Paper to Compile the “Voices From the Field.”. Qualitative Health Research. 2003; 13(2): p. 281–286. doi: 10.1177/1049732302239605 12643034

[100] Denzin NK, Lincoln Y, editors. Handbook of qualitative research London: Sage; 1994.

[101] Gioia DA, Pitre E. Multiparadigm Perspectives on Theory Building. 1990; 15: p. 584–602.

[102] Rashid M. The question of knowledge in evidence-based design for health care facilities: limitations and suggestions. Health Environments Research & Design. 2013; 6(4): p. 101–126. 10.1177/193758671300600407 24089184

[103] Dai Z, Liao X. Hospital-based health technology assessment: The next frontier for traditional Chinese medicine hospitals. Journal of Traditional Chinese Medical Sciences. 2021;(8): p. 110–114.

[104] Arab-Zozani M, Pezeshki MZ, Khodayari-Zarnaq R, Janati A. Balancing Overuse and Underuse in the Iranian Healthcare System: A Force Field Theory Analysis. Ethiopian journal of health sciences. 2019; 29(2). doi: 10.4314/ejhs.v29i2.10 31011271 PMC6460443

[105] Swanson DJ, Creed AS. Donald James Swanson; Andrew Shawn Creed; Sharpening the Focus of Force Field Analysis. Journal of Change Management. 2014; 14(1): p. 28–47.

[106] Shirey MR. Lewin’s theory of planned change as a strategic resource., 43(2),. JONA: The Journal of Nursing Administration. 2013; 43(2): p. 69–72. doi: 10.1097/NNA.0b013e31827f20a9 23343723

[107] Hussain ST, Lei S, Akram T, Haider MJ, Hussain SH, Ali M. Kurt Lewin’s change model: A critical review of the role of leadership and employee involvement in organizational change. Journal of Innovation & Knowledge. 2018;(3): p. 123–127.

[108] May C. A rational model for assessing and evaluating complex interventions in health care. BMC Health Services Research. 2006; 6(86). doi: 10.1186/1472-6963-6-86 16827928 PMC1534030

[109] Fisher MP, Hamer MK. Qualitative Methods in Health Policy and Systems Research: A Framework for Study Planning. 2020; 30(12): p. 1899–1912. 10.1177/1049732320921143 32449451

[110] Kooiman J, editor. Modern Governance. New Government-Society Interaction London: Sage Publication; 1993.

[111] Stoker G. Governance as theory: Five propositions. International Social Science Journal. 1998;(50(1)): p. 17–28.

[112] Poder TG, Bellemare CA, Bédard SK, Fisette JF, Dagenais P. Impact of health technology assessment reports on hospital decision makers–10-year insight from a hospital unit in Sherbrooke, Canada: impact of health technology assessment on hospital decisions. International journal of technology assessment in health care. 2018; 34(4): p. 393–399. doi: 10.1017/S0266462318000405 30021663

