# Supplementary material for: HB-HTA as an implementation problem in Polish health policy
Source: PLoS One. 2021 Sep 24;16(9):e0257451. doi: 10.1371/journal.pone.0257451 (PMC8462719; doi:10.1371/journal.pone.0257451)
Supplement: S1 File — The full list of identified driving and restraining forces. (PDF) [file pone.0257451.s001.pdf]

S1 File – Matrix of identified forces. The full list of identified driving and restraining forces

PEST		Force Field Analysis			
		Driving forces		Restraining forces	
**PEST dimension**	**Determinants/factor for dimension**	**Force field analysis Step1**<br>Assigning 7 driving forces to each PEST factor (total: 176 forces)	**Force field analysis Step2**<br>Reduction to 5 driving forces to each PEST factor (total: 140 forces)	**Force field analysis Step1**<br>Assigning 7 restraining forces to each PEST factor (total: 176 forces)	**Force field analysis Step2**<br>Reduction to 5 restraining forces to each PEST factor (total: 140 forces)
**POLITICAL**	**Building a political agenda**	Correct identification of key players		Lack of ability to prepare or failure to conduct identification of organizational and social actors and key players who will participate in the change implementation process	
		Rationally set goals for change		Expectations too high or too low about the results of the change implemented	**Reduced**
		Prepared from a resource-based perspective (RBV), a strategy for implementing change	**Reduced**	Inadequate choice of change strategy for existing resources (evolutionary strategy versus revolutionary/radical strategy)	

		An information package that takes into account the needs of diverse stakeholders		Incorrect identification of stakeholders and their information needs	
		Select channels for communicating information about the change and ways to implement it adequately to the identified audiences and their information needs	**Reduced**	Incompetent selection of ways and channels to communicate the prepared change and its potential outcomes	
		Preparation of alternative scenarios in case of crisis situations, e.g. increased resistance to accept the change goals		Failure to apply lessons learned from organizational learning, especially the double and triple loop of learning	**Reduced**
		Monitoring the change implementation process		Failure to apply organizational control and process staging principles	
	**regulatory capabilities**	Legislation governing the health care system	**Reduced**	Inconsistency of the existing law with regard to particular aspects of functioning of the health care system	
		Control of the conditions for the emergence of new regulations, especially their compliance with existing norms and the ways of proceeding with new regulatory solutions		Incomplete execution of the Regulatory Impact Assessment and the assessment of consequences of the introduction of a new solution	
		Rationality and functionality of the proposed solution for the placement of HB-HTA in the existing health care system		Failure to define legal and economic conditions for the introduction of HB-HTA into the health care system	**Reduced**
		Clear identification of tasks and authorities governing health technology assessment		Lack of prepared implementation in terms of administrative procedures, i.e. templates of documents, rules of procedure, evaluation criteria, ways of financing the selected solutions	
		Procedure for issuing marketing authorizations for medical procedures evaluated under HB-HTA and monitoring the results	**Reduced**	No or unclear sanctions for negative implementation results	**Reduced**

		Correctly identify the entities involved in the legislative process		Neglect of proper preparation of the policy agenda and the consequent blurred responsibility for preparing and carrying out the legal basis for the implementation and operation of HB-HTA	
		Working with stakeholders in the legislative process and building a support network		Underestimation of the role of collaboration and networking in the preparation and procedural phase of the HB-HTA regulatory model	
	**Implementation possibilities**	A properly prepared policy agenda for implementing HB-HTA and implementing legislation		Failure to prepare or careless preparation of dialogue instruments between all institutions involved in implementation	**Reduced**
		Identified resources that can be used effectively when implementing the new solution	**Reduced**	Failure to properly identify the resources necessary for effective implementation	
		Identify key stakeholders and their influence and importance to the success of the implementation	**Reduced**	Lack of ability to conduct stakeholder analysis and, on this basis, to draw conclusions about the directions and scope of information policy	
		Correct identification of competencies, tasks and roles of institutions operating in the hospital environment that will be involved in the HB-HTA implementation process		Lack of transparency and functionality of entities operating in the macroeconomic and task environment of the hospital	
		Establish incentive mechanisms for hospitals that implement HB-HTA		Lack of solutions to promote the introduction of HB-HTA units into the hospital structure	
		Expanded risk management practices as a formal requirement for the smooth operation of hospitals that choose to implement HB-HTA		Lack of risk management standards in hospitals	

		Effective communication in the planning, implementation and monitoring and control phases		Using communication channels that are inadequate to meet stakeholder needs	**Reduced**
	**Involvement of central institutions in promoting the introduction of HB HTA into the healthcare system**	Correct identification of health care policy objectives	**Reduced**	Lack of strategic vision and identification of strategic objectives in actions taken	**Reduced**
		Using EBM and EBPH to evaluate the potential outcomes of planned implementation		Intuitive rather than scientific preparation of the factual basis for the change undertaken in hospital organizational practices	
		Linking demographic, technological and medical trends to the need for new solutions		Failure to address emerging population needs in the proposed vision for HB-HTA implementation	
		Conduct an inventory of resources needed to implement HB-HTA	**Reduced**	Lack of in-depth understanding of available resources and allocation options	**Reduced**
		Use the knowledge, experience and expertise of international institutions and expert groups in the design phase of HB-HTA implementation		Incremental and political rather than merit-based preparation of HB-HTA implementation	
		Choosing an HB-HTA Implementation Model		Failure or unreliable evaluation of the effects of the proposed (central/regional) HB-HTA model	
		Preparation of organizational architecture diagrams of institutions supporting the implementation and identification of their roles and tasks		Lack of a transparent and clear organizational vision for the structure supporting HB-HTA implementation	
	**Involvement of local and regional institutions in promoting the introduction of HB**	Selected HB-HTA implementation model - regional		Implementing a central HB-HTA model	**Reduced**
		Priorities of regional health care policy	**Reduced**	Lack of strategic vision and identification of strategic objectives of regional health care policy	**Reduced**

	**HTA into the healthcare system**	The economic condition of the region and the public institutions in it	**Reduced**	Low GDP in the region, low quality of human capital employed in public institutions	
		Good financial condition of hospitals operating in the region		Poor financial condition of hospitals	
		Involvement of ownership entities in HB-HTA implementation		Politicking in place of real involvement of ownership entities in HB-HTA implementation	
		Preparation of organizational architecture diagrams of institutions supporting implementation and identification of their roles and tasks at the regional level - coordination		Lack of regional coordination	
		Networking of service providers		Reluctance to create regional inter-institutional networks	
	**Hospital independence, conditions, and adaptability**	Regulation of hospital autonomy		The actual autonomy of hospitals and the role of ownership entities	**Reduced**
		Use of EBM and EBPH in the practice of formulating health care policy goals	**Reduced**	Financial condition of hospitals	
		Quality of human capital and motivation of hospital employees	**Reduced**	Low level of employee motivation	**Reduced**
		Management's management skills		Old patterns of hospital management	
		Decision-making process and its determinants		Intuitive versus rational models for making financial allocation decisions	
		Risk appetite in operations and risk assessment standards		Low propensity to take risks in business	
		Flat structures (*lean management*) and organizational learning		Hierarchical structures, poor internal communication patterns	
	**Monitoring and evaluation**	The need to adapt to the requirements formulated in the EU strategic documents		Poor link between programme documents and strategic EU documents	**Reduced**
		Sector policy standards	**Reduced**	Low quality of legislation	**Reduced**

		Practices for monitoring the results of planned activities		Intuitive rather than evidence-based formulation of health care policy goals	
		Adopted procedures for evaluating the decision-making process	**Reduced**	Weakness of the decision-making process	
		Support solutions that promote the measurement of action results		Failure to promote performance measurement in organizational culture	
		Creating instruments that reward hospitals that use transparent methods to evaluate their performance		Disregarding the results presented in audit documents and failing to review its own activities	
		Activities aimed at developing managerial competencies among hospital managers		Reluctance to develop knowledge and competence in management, especially risk management	
**Economic**	**Expenditure on health care**	Bridging the gap with other EU member states		Budgetary constraints	
		The needs of an aging population		An ageing society	
		Implementing the welfare state		Emigration of young people	
		Pressure from hospital founders		Adverse economic situation	
		Patient pressure		Declining stock of economically active people	
		Rising medical technology costs	**Reduced**	Public dissatisfaction with contribution rates	**Reduced**
		Pressure from medical services	**Reduced**	Decreasing EU funds	**Reduced**
	**The share of public expenditure on health care of total health expenditure**	Pressure to "catch up" with Western European countries		Budgetary constraints	
		Prosperity		Economic downturn	
		Pressure to spread/improve access to benefits		Other urgent and important budget expenditures	
		Pressure to improve the quality of benefits		Reduced premium revenue due to outflow of workforce	
		Availability of EU funds	**Reduced**	Reduced premium revenue due to an aging population	

		The growing health needs of an aging population		Outflow of patients to private providers	**Reduced**
		Health as a priority goal of the state	**Reduced**	Health packages offered by employers	**Reduced**
	**The share of expenditure on salaries of the total hospital costs,**	Outflow of medical workers abroad		Current expenses	
		The need to increase employee motivation		Spending on investment in new technologies	
		Public pressure for high quality medical service		Research funding	**Reduced**
		Closing the gap with the EU average (earnings)		Need to maintain existing infrastructure	
		The need to advance medical research		Budgetary constraints	
		Trade union pressure	**Reduced**	Patient pressure on drug reimbursement	**Reduced**
		The need to increase employee motivation	**Reduced**	Patient pressure to invest in new equipment	
	**Costs of medical technologies**	The need to achieve EU standards		Employee pressure to increase wages at the expense of investment	
		Pressure on drug reimbursement		Budgetary constraints	
		Technological progress		Limitations on lump sum benefits	**Reduced**
		Pressure on service quality		Expenditure on maintenance of existing infrastructure	
		Replacing old technologies with new ones	**Reduced**	Lack of access to comprehensive knowledge about new technologies	
		The need for high quality research	**Reduced**	Limited ability to assess the profitability of introducing new technologies	**Reduced**
		Lobbyists supporting medical corporations		Risk of failure when introducing new technologies	
	**Availability of European funds for investments in**	Health as a priority for the EU		Inability to obtain EU funds	
		Access to new infrastructure		The need to commit to an own contribution	

	**innovative medical technologies**	Support the new Member States in bridging the socio-economic gap		Low quality of research compared to EU average	
		Growing health care needs of the population		Outflow of scientists and medical personnel (emigration)	
		Growing experience in obtaining EU funds		Decreasing resources of European funds	
		Pressure to raise EU funds due to declining subsidies	**Reduced**	Discouragement with lack of success in obtaining EU funds	**Reduced**
		Pressure to raise EU funds due to the spectre of crisis in the EU	**Reduced**	Mismatch between the offer of EU funds and the diverse needs of Polish hospitals	**Reduced**
	**Flat-rate financing of hospitals**	HB-HTA as a facilitator of benefits optimization		Insufficient funds for investment	
		More accurate information on the cost-effectiveness and medical effectiveness of applied technologies with HB-HTA		Additional responsibilities for employees	
		Increasing cost efficiency		With lump sum funding, it is easier to manage repetitive procedures than to introduce new ones	
		Better financial management of hospitals		Lack of funds for additional pay for employees	
		More freedom for hospitals to choose the technologies they use		Difficulty in obtaining funding for new technologies	
		Ability to tailor services to the needs of your patients	**Reduced**	Too fast another change in hospital management	**Reduced**
		Increased medical efficiency	**Reduced**	Low adaptability of hospitals to change	**Reduced**
	**Competence of hospital management in HB-HTA analysis skills**	Greater decision-making independence for hospital managers		Lack of funding for additional salaries for HB-HTA implementation staff	
		Opportunity to acquire new competencies		Lack of adequate staff	
		Opportunity to receive a raise	**Reduced**	Need to make new hires	
		Publicly funded training		Lack of clear guidance on HB-HTA operating procedures	

		The emergence of tailored educational offerings at universities		Reluctance of managers to take on new tasks	
		Possible incentives (e.g., financial) for managers		Managerial overwork/burnout	**Reduced**
		Advancement opportunities	**Reduced**	Disagreements/ friction among staff	**Reduced**
**Social**	**Patient participation in the process of assessing hospital performance**	Educate patients about effective treatment options		Lack of knowledge among patients about effective treatments	**Reduced**
		Improving the quality of medical and preventive services		Hierarchical basis of the relationship between patient and medical professional	
		Transparency of treatment results made available by the hospital	**Reduced**	Lack of participation and co-production in the design of health services	
		Easy access to information about innovative treatment methods used by the hospital		Limited access to information about medical technologies used in the hospital	
		Preparation of procedures and patient participation in evaluating hospital outcomes	**Reduced**	Underdeveloped procedures of evaluation of non-economic results of hospital activities by local government bodies	
		Feedback between medical procedures used by the hospital and community quality of life		Low propensity of hospitals to disseminate treatment outcome information	
		Making hospital financial support dependent on the results achieved, including the use of modern technologies		Claiming attitudes among patients	**Reduced**
	**Pressure of service recipients on prevention and effective and minimally invasive treatment**	Growing public awareness of their own health needs		Late or limited access screening tests	
		Development of minimally invasive methods of treatment and diagnosis		Overvaluing interventional medicine to the detriment of preventive and preemptive medicine	**Reduced**

	**methods improving the quality of life**	Engaging the private sector to retrofit their treatment entities	**Reduced**	Lack of effective education that shapes citizens' sense of responsibility for their health	
		Pressure to expand the range of services provided in the public sector	**Reduced**	Uneducated active pro-health attitudes	**Reduced**
		New fiscal formulas to create earmarked funds usable for health care		Lack of state involvement in creating mechanisms to incentivize the use of available prevention services	
		Development of population-based health programs		Underestimation of the outcomes of peri-medical professionals (e.g., public health graduates) by the health sector	
		Redirecting attention from restorative medicine to preventive medicine and health promotion		Inadequate directions for allocating money in the health care system to meet the challenges	
	**Aging of the population and pressure to increase the effectiveness of medical care**	Access to EU funds for population programs		Lack of ability to raise funds	
		Development of research on old age and its problems	**Reduced**	Wasting resources	
		The pressure to age actively and healthily		Cyber exclusion of older people	
		The need to respond to demographic change		Limiting access to modern medical technology for income reasons	
		Developing technological options to support older adults in a residential setting		Habits and habits of people in the older population to use traditional solutions	
		The rise of technology in elderly and dependent care	**Reduced**	Patients' Claims	**Reduced**
		Increasing political power of the elderly population - gerontocracy		Multi-morbidity making diagnosis difficult	**Reduced**
		Development of e-health		Limited implementation due to low digital competence of staff	

	**Competence of medical professionals in the use of modern medical and non-medical technologies**	The need for healthcare professionals to acquire digital competencies		Reluctance of patients and health care professionals to go deeper into the use of ICT	
		Changing age structure of medical professionals	**Reduced**	Staff shortages in the health care system	**Reduced**
		Development of healthcare functionality as a consequence of wider use of ICT	**Reduced**	Inadequacy of investment in innovative equipment to potential benefits (reimbursement problems)	**Reduced**
		Development of modular education in the use of digital technologies in diagnosis and treatment		Health professions market drain	
		EU legislation supporting the use of ICT		Quality and accessibility to postgraduate education	
		Availability of non-state funds for ICT development		Staffing shortages among the treatment support staff group (e.g., coordinator, secretary)	
	**Competence of managers of medical facilities in the field of implementing modern medical and non-medical technologies**	Pressure to manage effectively		Limiting hospital funding	
		Development of universal competences		Age and professional structure of hospital managers. Problems of leadership.	
		ICT-supported management processes		Low level of adaptability to changing environmental conditions	
		Changing the age structure of managers	**Reduced**	Lack of dedicated highly specialized staff for management in hospitals and other health care entities	
		Standardization of management processes		Lack of competent decision-making support	
		Use of strategic management instruments in the management process	**Reduced**	Low willingness to share experiences and knowledge	**Reduced**
		Benchmarking and knowledge diffusion		Low crosslinking	**Reduced**
		Development of medical tourism in the world and in Europe		Patient attachment to the home health care system.	**Reduced**

	**The share of the Polish healthcare sector of the medical tourism market.**	Formalization of interest in the phenomenon (service providers, institutions of central and local government)		Political and social tensions in the world and in Europe	
		EU regulations	**Reduced**	Lack of a comprehensive medical tourism policy strategy	**Reduced**
		Pressure for high quality of treatment in medical entities	**Reduced**	Too little (financial and in-kind) support for providers to develop medical tourism	
		Increased ability to search online resources for opportunities to access benefits		Scattered anecdotal information on medical tourism in Poland	
		Increased confidence in the Polish health care system		Lack of universal certification and accreditation of medical entities	
		Increased awareness of cross-border rights		Fading importance of EU directives on cross-border care	
**Technological**	**Digitization**	Requirements set by the European Union		Lack of standards for evaluating out-of-the-box conduct	
		Staff shortages		Attitude of the medical staff	
		Regulations		Rigid evaluation criteria in health technology assessment	
		Digitalization of society		Social awareness	**Reduced**
		Limited financial resources with rising public expectations		Lack of clear guidelines / definitions for digital technologies at HTA	**Reduced**
		Digitalization in the private healthcare sector	**Reduced**	Lack of funding in district and community hospitals	
		IT technology development	**Reduced**	Inadequate IT infrastructure	
	**Telemedicine**	Ageing of the population		Limited public funding for telemedicine services	
		Regulations	**Reduced**	Social awareness	
		Staff shortages		Standardization of the evaluation of out-of-the-box procedures in HTA	**Reduced**

		Requirements set by the European Union		Insufficient support for telemedicine in preventive health programs of TSU	
		Digitalization of society	**Reduced**	Inconsistency of regulations	**Reduced**
		Difficult access to a doctor / limits		Inadequate IT infrastructure	
		Digitalization in the health care system		Awareness of primary care physicians	
	**Artificial intelligence**	Staff shortages		Public awareness	**Reduced**
		Digitalization of society	**Reduced**	No HTA coordinator	**Reduced**
		Ageing of the population		Lack of financial resources in the health care system	
		Development of AI technology	**Reduced**	Resistance from doctors	
		Digitalization in health care		Inadequate infrastructure	
		Increased ability to use modern technology among medical professionals		Regulations	
		Public expectations regarding the quality of health services		Lack of knowledge and competence of management	
	**Personalized medicine**	Public expectations about the quality and effectiveness of health services		Benefit costs	
		Increase in the incidence of cancer		Regulations	
		Digitalization in health care	**Reduced**	Limiting access to molecular diagnostics	
		Knowledge dissemination among physicians and patients	**Reduced**	Ethical issues	**Reduced**
		Development of drug technologies		Preference for traditional methods	**Reduced**
		Value Based Healthcare		Lack of certification and accreditation system for genetic testing providers	
		Advances in genetic knowledge		Medical degree programs	

	**Development of new drug technologies**	Ageing of the population		Lack of a national strategy for the pharmaceutical industry	**Reduced**
		Development of personalized medicine		Regulations	**Reduced**
		New health challenges		Low national investment in new therapies	
		Value Based Healthcare		Long time to market for the drug	
		European Union	**Reduced**	Downward pressure on drug prices from the Ministry of Health	
		Scientific Potential		Procrastination of administrative procedures in the implementation of clinical trials	
		National Center for Research and Development / StrategMed	**Reduced**	Low share of health expenditure in GDP	
	**Development of new non-drug technologies**	Rapid development of modern technologies		Financial situation of medical entities	
		Expectations for higher quality medical devices from the medical professional community		Procedure for introducing new medical devices into the basket of guaranteed benefits	
		Ageing of the population		Bureaucracy in bringing medical devices to market	
		National Centre for Research and Development	**Reduced**	Limited knowledge of medical professionals and patients regarding new non-drug technologies	
		Value Based Healthcare		Diversity of public funding schemes	
		Well-educated medical and engineering staff		Lack of data necessary to perform HTA at the hospital level	**Reduced**
		Medical Research Agency	**Reduced**	The rapid pace of new product launches	**Reduced**
	**The Agency for Health Technology Assessment and Tariff**	Formal sanctioning of cooperation		Fear of limiting AOTMiT's competence	
		Digitalization in health care		Lack of interest of the parties in establishing cooperation	**Reduced**

	**System technological support**	Project "Rational decisions in the health care system, with particular emphasis on regional health policy"		Lack of funding source for cooperation	
		The financial incentive to use HB-HTA		Imbalance in the relationship	
		Establishment of a network of regional branches of AOTMiT		Different needs for HTA	
		Partnership and trust in relations between AOTMiT and hospitals	**Reduced**	Concern about conflicting assessments of the same technology in national and hospital HTA	
		Implementation of joint projects	**Reduced**	Lack of management commitment and competence	**Reduced**
	**Creation of a cooperating network in the field of HB-HTA**	Digitalization in health care		Competition between hospitals	
		Reduction of analysis costs		Lack of collaborative culture	**Reduced**
		Relationships based on partnership and trust	**Reduced**	Lack of management commitment	**Reduced**
		Joint research projects	**Reduced**	Relationship imbalance	
		Financial incentives for using HB-HTA		Central impositions of network creation	
		Formalization of networks		Physicians' perceptions of loss of decision-making autonomy	
		Joint development of guidelines for HB-HTA		Costs of network participation	
**Researchers:**<br>Female, Ph.D, subject of intrests: management, healthcare policy, HTA<br>Female, Ph.D, Prof. subject of intrests: health sciences, political sciences, healthcare policy, HTA		**Researchers:**<br>Female, Ph.D, Prof. subject of intrests: management, political sciences, public management, healthcare policy, HTA<br>Female, Ph.D, subject of intrests: economy, public policy, global social policy<br>Female, Ph.D, subiect of intrests: management, healthcare public management, accounting			

Female, Ph.D, Prof. subject of intrests: management, political sciences, public management, healthcare policy, HTA<br><br>Female, Ph.D, Prof. subject of intrests: economy, healthcare policy, HTA<br><br>Female, Doctor of MD, subject of intrests: medicin, health policy, HTA<br><br>Male, Doctor of MD, subject intrests: medicine, healthcare systems, HTA<br><br>Female, M.Sc. subject of intrests: pharmaceutical sector and health care institutions, HTA<br><br>Male, M.Sc. subject of intrests: technology development, software engineering, health policy, HTA<br><br>Female M.Sc. subject of intrests: analysis of the market and trends in the health sector in the context of the development and implementation of new technologies, HTA<br><br>Female, M.Sc. subject of intrests: healthcare management, HTA<br><br>Male, M.A. subiect of intrests: health economics and health governance, HTA	
